# On the Thermodynamic Thermal Properties of Quercetin and Similar Pharmaceuticals

**DOI:** 10.3390/molecules27196630

**Published:** 2022-10-06

**Authors:** Costas Tsioptsias, Ioannis Tsivintzelis

**Affiliations:** Department of Chemical Engineering, Aristotle University of Thessaloniki, University Campus, 54124 Thessaloniki, Greece

**Keywords:** thermochemical transition, melting, decomposition, flavonoids, hydrogen bonding, quercetin

## Abstract

The thermodynamic properties of pharmaceuticals are of major importance since they are involved in drug design, processing, optimization and modelling. In this study, a long-standing confusion regarding the thermodynamic properties of flavonoids and similar pharmaceuticals is recognized and clarified. As a case study, the thermal behavior of quercetin is examined with various techniques. It is shown that quercetin does not exhibit glass transition nor a melting point, but on the contrary, it does exhibit various thermochemical transitions (structural relaxation occurring simultaneously with decomposition). Inevitably, the physical meaning of the reported experimental values of the thermodynamic properties, such as the heat of fusion and heat capacity, are questioned. The discussion for this behavior is focused on the weakening of the chemical bonds. The interpretations along with the literature data suggest that the thermochemical transition might be exhibited by various flavonoids and other similar pharmaceuticals, and is related to the difficulty in the prediction/modelling of their melting point.

## 1. Introduction

Quercetin belongs to the flavonols, one of the main classes of the flavonoids. As for all pharmaceuticals/nutraceuticals, their thermodynamic properties, such as their melting points, heats of fusion and heat capacities, are important for understanding and predicting drug absorption [1], optimizing drug processing, and modelling and developing new drugs [2,3]. However, it has been recognized that the modelling/predicting of properties, such as the melting point, is difficult to achieve in various pharmaceuticals [2,4]. This probably suggests a lack of a complete understanding of the thermal behavior of such pharmaceuticals.

For some solids, already decades ago, an inability for actual melting (that is melting without decomposition) has been observed. For example, it is known that polysaccharides, such as chitin and cellulose [5], do not exhibit any thermal transition prior to decomposition, similarly to various proteins, e.g., gelatin [6]. Recently, it was recognized that the thermal transitions of cellulose esters [7,8] and poly (vinyl alcohol) (PVA) [8,9], erroneously attributed to melting, were observed due to a peculiar effect of simultaneous material’s softening and decomposition. The term “glass chemical transition” was initially proposed [7], and after recognizing its broadness, the term “thermochemical transition” [8] was introduced to describe this unique effect. Although the quantitative extent of decomposition during softening is different, e.g., in cellulose and PVA, the behavior is qualitatively the same, i.e., an inability for actual melting without decomposition. Besides polymers, similar behavior has been sporadically observed for some low molecular weight substances, such as lithium potassium tartrate [10], potassium perchlorate [11] and flavonoids such as quercetin dihydrate [12] and rutin dihydrate [12]. In addition, variation in the reported values of succinic acid’s melting point has been suspected to arise from the decomposition and/or acid dehydration and formation of anhydride upon heating [13]. Very recently, the thermal transition of the flavonoid silybin was recognized as a thermochemical transition, which was erroneously considered as melting [14]. For gallic acid, two thermochemical transitions were reported, namely a solid–solid and solid–liquid thermochemical transitions [15]. In addition, various explosives have melted during decomposition, e.g., HMX (Octahydro-1,3,5,7-tetranitro-1,3,5,7-tetrazocine) [16]. It is apparent that the vast majority of substances exhibiting a thermochemical transition are substances with the capability for increased hydrogen bonding.

Bawn kinetics is a branch of chemical kinetics focusing on the decomposition time profile of solids, for which some melting occurs during their decomposition, and how this profile is influenced by the formation of the liquid phase [17]. The Bawn model has been used for various explosives, and also for various pharmaceuticals [17]. Although the Bawn model was developed decades ago, to the best of our knowledge, no connection has been made with the peculiarities in the thermal (thermodynamic) transition behavior of pharmaceuticals [12], since such a model is a decomposition kinetic model. In addition, no connection of these two concepts (peculiarities in the thermal behavior of pharmaceuticals that are observed experimentally, and the fact that Bawn kinetics have been used for pharmaceuticals) with the difficulties [2,4] in predicting/modelling of a pharmaceutical’s melting point has been made. The lack of a connection among the above three aspects, probably, should be attributed to the lack of a complete understanding of the thermal behavior of some pharmaceuticals, which also seems to be the main reason for the confusion in the literature regarding the thermodynamic properties of such compounds, especially their melting point. In what follows, we briefly attempt to highlight this confusion.

One recognized factor for this confusion is the existence of various polymorphs with different degrees of hydration, e.g., for quercetin [18]. Three different polymorphs of quercetin hydrates have been reported to exhibit losses of endocrystalline solvent (water) at 82, 105 and 119 °C, and to exhibit melting at temperatures of 317, 318 and 323 °C [18]. In addition, for the same polymorphs, the occurrence of decomposition at temperatures between 340 and 350 °C, slightly higher than the melting temperature, was reported [18]. For two of these polymorphs, a solid–solid transition was speculated to occur in order to explain the small endothermic peaks observed, prior melting, at 231 and 272 °C [18]. Similarly, for quercetin dihydrate (and rutin dihydrate) multiple transitions were detected [12]. Precisely, for quercetin dihydrate it was reported that no melting occurred prior decomposition and that at 116 °C decomposition related to water loss occurred [12]. In other studies for quercetin, various similar values have been reported for the melting point, e.g., a melting point at 315 °C with a heat of fusion of 51.08 kJ/mol along with glass transition at 108 °C [19], a melting point of 326.7 °C [20], a melting point of 322 °C with a heat of fusion of 41.5 kJ/mol [21], and, very recently, a melting point of 323 °C [22]. As a summary, the transitions of quercetin at low temperatures (around 100 °C) have been attributed to various different effects, such as dehydration/decomposition or glass transition, while the transition at higher temperatures (around 320 °C) is typically attributed to melting. However, decomposition has been realized to occur slightly above melting, and solid–solid transitions prior to melting were speculated to occur. The aim of this work is to clarify this confusion about the thermal behavior of quercetin and to show that its peculiar thermal behavior is, most likely, exhibited by other similar molecules.

## 2. Experimental

Quercetin, solid (powder), with purity >95% (HPLC), was purchased from Sigma Aldrich (St. Louis, MO, USA). Potassium bromide (KBr) with purity >99.5 wt.% was purchased from Chem-Lab. A Shimadzu TGA-50 thermogravimetric analyzer, a Shimadzu DSC-50 calorimeter, a Biorad FTS-175 spectrometer, a USB digital microscope and a Brucker (model D8 Advance) equipped with a Siemens X-Ray tube (Cu, 1.54 Å) diffractometer were used for the thermogravimetric analysis (TGA), the differential scanning calorimetry (DSC) measurements, the Fourier transform infrared spectroscopy measurements (FTIR), for the observation of the samples prior to and after heating, and the X-ray diffraction measurements (XRD), respectively. The samples were weighted with a Sartorius scale (model B 120S, ±0.0001 g).

The DSC measurements of quercetin were performed under a nitrogen atmosphere (flow rate of 20 mL min^−1^) with a heating rate of 10 K min^−1^ from 40 to 350 °C. Three TGA measurements of quercetin were carried out under a nitrogen atmosphere (flow rate of 20 mL min^−1^) with a heating rate of 10 K min^−1^ from 40 to 600 °C, from 40 to 320 °C, and from 40 to 135 °C. The sample heated in the TGA at 320 °C was observed under a microscope. The sample heated up to 135 °C and a raw quercetin sample, were observed under a microscope and were studied by FTIR and XRD. The powder samples were mixed with KBr at a mass proportion of 1:200 (sample mass to mass of KBr) and processed into pellets with a hydraulic press (100 Bar). Before using, the KBr was air dried for 3–4 h at 140 °C. For the FTIR measurements, 64 scans were taken in absorption mode at a resolution of 2 cm^−1^.

## 3. Results and Discussion

**Figure 1:**
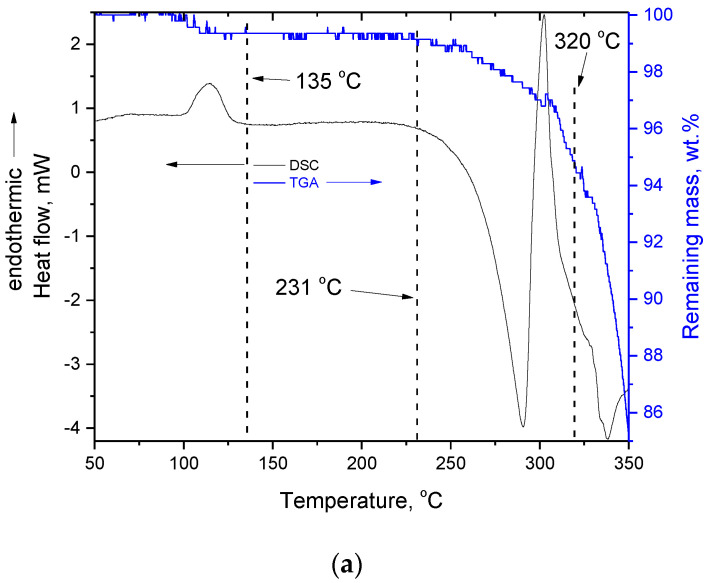

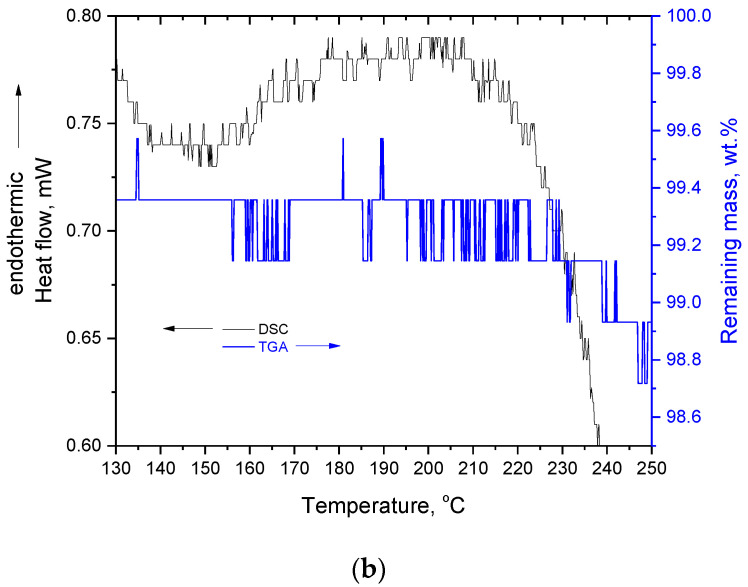
DSC and TGA curves of quercetin: (**a**) in the temperature range 50–350 °C, and (**b**) under different scale in the Y-axes and in the temperature range 130–250 °C.

The DSC and the TGA curves of quercetin, in the temperature range of 50–350 °C, are presented in Figure 1a. It should be stressed that the noise arises from the instability of the signal and is equal to the readability/sensitivity of the instruments (±0.01 mW for the DSC and ±0.01 mg for the TGA), and does not interfere with the evaluation of the respective curves (in the TGA results, it only increases the uncertainty for the mass loss). In addition, it is worth mentioning that the DSC signal is not stable until the same temperature is reached in both pans, i.e., sample and reference. In order to exclude this (non-interpretable) signal alteration, the DSC data up to 50 °C were not taken into account. Thus, the TGA data in Figure 1a are presented from 50 °C.

As can be seen in the DSC curve (Figure 1a), in the temperature range 90–135 °C a small endothermic peak is detected and is obviously related to the mass loss of 0.86 ± 0.33 wt.% detected in the TGA curve. As discussed in the introduction, this effect has been attributed to a glass transition, or dehydration and decomposition related to water loss. Both the heat flow (the DSC signal) and the mass loss (the TGA signal) are stable beyond approximately 135 °C. The TGA signal is stable up to approximately 230 °C, however, the DSC signal remains stable only for a small temperature range (135–150 °C), and then it increases. A small, broad endothermic peak in the range of 150–230 °C can be detected under a different Y-axis scale (Figure 1b).

**Figure 2:**
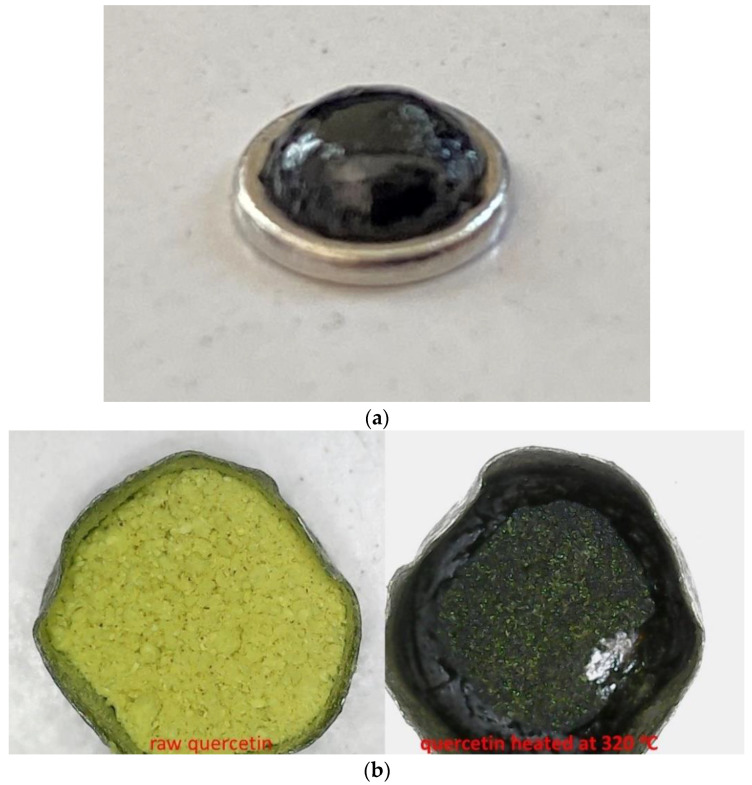
(**a**) Photograph of the DSC pan after the measurement up to 350 °C showing that mass loss out of the pan occurred during the measurement, and (**b**) Photographs from digital microscope of quercetin in the TGA pan before and after being heated at 320 °C.

This small peak most likely is related to the reported solid–solid transition of some quercetin polymorphs [18], however, it cannot be a thermophysical transition, as will now be discussed. It is apparent from the TGA curves in Figure 1 that the decomposition initiates at 230 °C. This mass loss is also detected by the DSC after 230 °C. More precisely, the DSC signal is influenced by the heat capacity of the sample (mass times specific heat capacity) and by any endothermic, or exothermic, phenomena. Endothermic effects and any increases in heat capacity shift the DSC signal towards the endothermic direction, while a decrease in heat capacity, or any exothermic effect, shifts the signal to the opposite direction. After 230 °C, the DSC signal shifts intensively towards the exothermic direction, and this is caused by the decrease in the heat capacity, due to the decrease in the sample mass. It is stressed that the DSC pans used in this study cannot stand high pressure and thus the actual mass loss out of the pan occurs as it is evident from Figure 2a. If the DSC pans had been hermetically closed, then the produced vapor phase would not have had such a tremendous influence on the DSC signal (this is mentioned because in the literature this intense exothermic shift has rarely been detected). Thus, both TGA and DSC detected mass loss after 230 °C. This mass loss was obviously related to decomposition, consequently, the following question arises: Where is the heat required for the mass loss after 230 °C? The small endothermic peak in the range 150–230 °C is the only thermal effect that could be attributed to the heat required for the initiation of decomposition. We performed a separate TGA measurement up to 240 °C and (macroscopically) no particle coalescence could be observed (photo not shown), thus, we confirmed that no liquefaction occurred. However, this solid–solid transition [18], detected as an endothermic peak in the DSC curve, is related to the initial stage of decomposition. It is worth mentioning that the area of the peak is small and thus corresponds to a small amount of heat. However, from Figure 1b it is obvious that there is no mass loss in the temperature range of this minor peak, or more accurately, there is no detectable mass loss in the TGA. As in all analytical techniques, in TGA there is also a limit of detection for the mass loss. In other words, if the small amount of heat of the endothermic peak of 150–230 °C could be expressed as the specific heat of a thermochemical transition [9], then it would be divided by a very small (not detectable in our case) amount of decomposed mass. Thus, the specific heat corresponding to this peak should be high. In addition, as reported recently [9], in the specific heat of a thermochemical transition there is a contribution of the activation energy for the initiation of decomposition. Thus, the peak in the range of 150–230 °C most likely includes the activation energy and the heat required for the initiation of decomposition. As the decomposition proceeds, the structure of the decomposed residue is different than that of untreated quercetin. Obviously, the new structures require different activation energy and additional heat for further decomposition. These are embedded in the intense peak at 303 °C. In addition, it is worth mentioning that despite the fact that the absolute areas of the small peak around 210 °C and the intense peak around 303 °C are quite different, the corresponding mass loss is also quite different. Thus, if these are expressed as specific heats, most likely, values of the same order of magnitude would be obtained (for the peak at 303 °C this calculation is possible and is discussed next).

During the continuous mass loss after 230 °C, the DSC signal shifts and, as already mentioned, an intense endothermic peak is detected with a maximum at 303 °C. This endothermic peak has been commonly (erroneously) attributed to the melting of quercetin (see Introduction section). This endothermic peak seems to end at approximately 320 °C and for this reason a separate TGA measurement was performed up to 320 °C. A photograph of this sample, along with a photograph of the raw quercetin sample, are presented in Figure 2b. From the obvious shrinkage and change in color, along with the mass loss detected in the TGA, it is clear that quercetin does not exhibit any actual neat melting (melting without decomposition). It is worth mentioning that the DSC signal, at around 340 °C, again shifts towards the endothermic direction. Consequently, a combined interpretation of the DSC and TGA curves in the range 150–350 °C leads to the conclusion that quercetin does not simply melt, but decomposition and structural relaxation/softening/“melting” occur at overlapping ranges. The DSC curve is actually the result of various consecutive overlapping signal alterations, due to the heat absorbed for the decomposition of quercetin, and its decomposed residue (endothermic shifts/peaks of the DSC signal) and a mass loss (exothermic shift of the DSC signal).

For quercetin dihydrate, it has been reported that the endothermic peak around 100 °C is due to decomposition related to water loss [12]. In this temperature range, the mass loss of quercetin dihydrate was 7–8 wt.%, and at 130 °C fluidization occurred [12]. This behavior is pretty similar to the one that has been reported recently for silybin [14], thus, this effect in quercetin dihydrate seems to be a thermochemical transition. The quercetin used in the current study is not the dihydrate form and exhibits a much lower mass loss (0.9 ± 0.3 wt.%) around 100 °C (these values were calculated from three independent repetitions of the TGA measurements). This could be easily attributed to water loss. However, since the dihydrate form has actually exhibited a thermochemical transition [12] in this temperature range, we checked if decomposition occurs also in the non-dihydrate form. For this purpose, two approaches were adopted: (1) an estimation of the specific heat for this effect, and (2) an investigation of the structure of raw quercetin and quercetin heated at 135 °C by FTIR and XRD.

The results for the calculations of specific heat for the two main endothermic peaks of quercetin (at around 100 and 303 °C) are presented in Table 1. For the peak at 303 °C, the specific heat was calculated with two different approaches, namely, the established way of heat of fusion (dividing the heat detected by the DSC by the total mass of the initial sample, a procedure that is followed if such a peak is attributed, erroneously, to neat melting), and the alternative way recently proposed [9] for the thermochemical transition (dividing the heat by the mass loss in the temperature range of the peak). For the peak around 100 °C, only the alternative approach has a physical meaning and will be presented. The value of the heat of “fusion” for the peak at 303 °C (69–75 kJ/mol) is of the same order of magnitude with reported values (~51 kJ/mol [19] and ~41 kJ/mol [21]). Of course, the physical meaningfulness of this value is highly questionable. For various reasons (not volatile decomposition products that are not detected as mass loss in the TGA and uncertainty of the heat measured by the DSC due to an alteration of the heat capacity baseline due to mass loss), which have been discussed recently in detail [9,14], the uncertainty for the specific heat of a thermochemical transition is quite high, and the presented values should not be considered as accurate values. On the contrary, more attention should be given to their order of magnitude. The uncertainty in the specific heat of thermochemical transition values, shown in Table 1, arises from the uncertainty of the mass loss measured by the TGA. In addition, in Table 1, wherever possible, the values of any specific heats (e.g., heats of fusion) are provided in both J/g and kJ/mol values. For the specific heat of thermochemical transitions, the values are expressed only in J/g because the molecular weights of the decomposition products are unknown.

Using the alternative approach, the value for the specific heat of thermochemical transition at around 300 °C (4517 J/g) is an order of magnitude higher than the heat of fusion value, and comparable to the chemical bond strength values [9]. Of similar order of magnitude is the specific heat for the peak around 100 °C (within the range 1865–4110 J/g), which is comparable and rather higher than the heat of the vaporization of water at 1 bar and 100 °C (~2257 J/g [23]). In addition, this value is quite higher than various reported ones for the heat of water desorption, which of course depends on the water content and reaches its maximum value in the monolayer region [24]. Such reported values for the heat of water desorption range from around 2 kJ/mol (~66 J/g) to 8 kJ/mol (~454 J/g) for desorption from lignite [24], from ~5 kJ/mol (278 J/g) to ~40 kJ/mol (~2222 J/g) for desorption from montmorillonites [25], and from ~2 kJ/mol (~111 J/g) to ~36 kJ/mol (~1983 J/g) for desorption from an apple [26]. Thus, the experimental value for the specific heat of the peak at 100 °C and its similarity with the value for the heat of the peak at 303 °C, where undoubtedly decomposition occurs, suggest that the mass loss at around 100 °C is probably not only a physical process, but chemical bond breakage is likely to occur.

**Figure 3:**
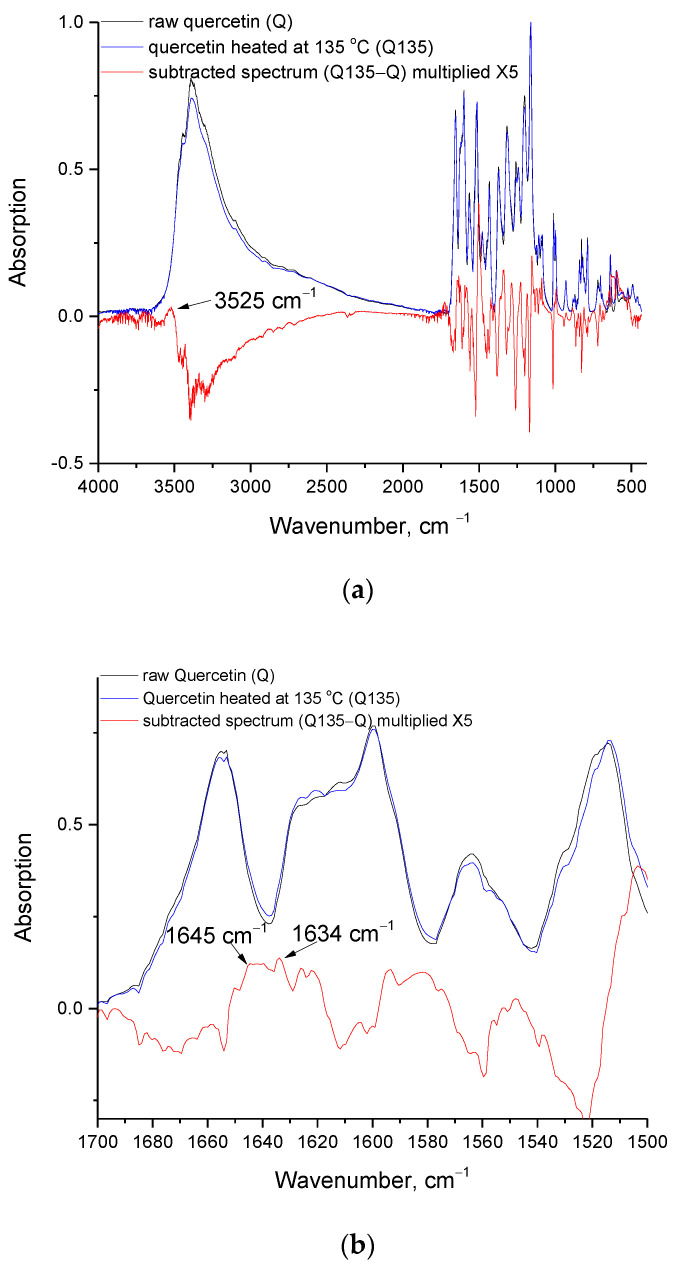
FTIR spectra of raw quercetin (Q) and quercetin heated at 135 °C (Q135) and their subtracted spectrum (Q135-Q) in the range: (**a**) 400–4000 cm^−1^, and (**b**) 1500–1700 cm^−1^.

FTIR seems to confirm this. As mentioned above, a separate TGA measurement up to 135 °C was performed, and the sample was studied by FTIR (along with a quercetin raw sample). At this temperature, both the TGA and DSC signals have stabilized (after the initial mass loss), and this is the reason for selecting such a temperature. Before discussing the FTIR spectra, it is worth mentioning that, in contrast to the dihydrate form [12], in our quercetin sample, no fluidization could be observed (photo not shown), since the softening during the thermochemical transition is decomposition-induced/assisted, and in this particular case, the decomposition occurred at a low extent, which is not adequate to cause a macroscopic softening. The FTIR spectra (Figure 3a) of the raw quercetin sample and the sample heated at 135 °C are pretty much alike, however, after baseline corrections and normalization, in their subtracted spectrum, various (negative and positive) peaks of different intensity are revealed (to increase visibility, the subtracted spectrum was multiplied by a factor of five). The normalization was performed with respect to the peak at 1160 cm^−1^ (-C-OH stretching vibration [27,28]). The main negative peak in the subtracted spectrum concerns the region 3000–3600 cm^−1^ (O-H stretching vibrations [29] and =C-H stretching vibrations in the range 3000–3100 cm^−1^ [29]). Interestingly, within this negative peak, a small positive peak at 3525 cm^−1^ can be detected (Figure 3a). It is known that the free OH groups vibrate at frequencies (wavenumbers) higher than the hydrogen bonded OH groups, and also that the phenolic OH vibrations occur in the range from 3500 to 3550 cm^−1^ [29].

Accordingly, this small positive peak is attributed to the free phenolic OH groups and suggests a slight increase in the free phenolic OH groups, after heating at 135 °C. In the negative peak appearing in the range of 3000 to 3500 cm^−1^ there are potential contributions from water, the phenolic bounded OH and =C-H groups. However, the contribution of water can be disputed based on the bending vibration of water, which occurs at 1645 cm^−1^ [29], or 1635 cm^−1^ [25]. There is an overlapping of these bands with the >C=O vibration at around 1650 cm^−1^ [27,29], however in the range 1620–1650 cm^−1^, a slightly positive absorption in the subtracted spectrum can be claimed. This suggests that the (relative) water content has not decreased. Consequently, the decomposition at 100 °C cannot be fully related to water removal, and an explanation would be that the water is endocrystalline and, thus, a portion of it is removed at temperatures higher than 100 °C. Thus, from the FTIR results it cannot be supported that the main, or exclusive, contribution to the negative peak in the OH stretching region arises from water removal. In addition, the C=O band at around 1670 cm^−1^ (as well other bands in the region 1500–1700 cm^−1^ as can be seen in Figure 3b) also are slightly negative and/or shifted and, thus, again, a loss of organic groups and an alteration of the chemical structure is indicated.

**Figure 4:**
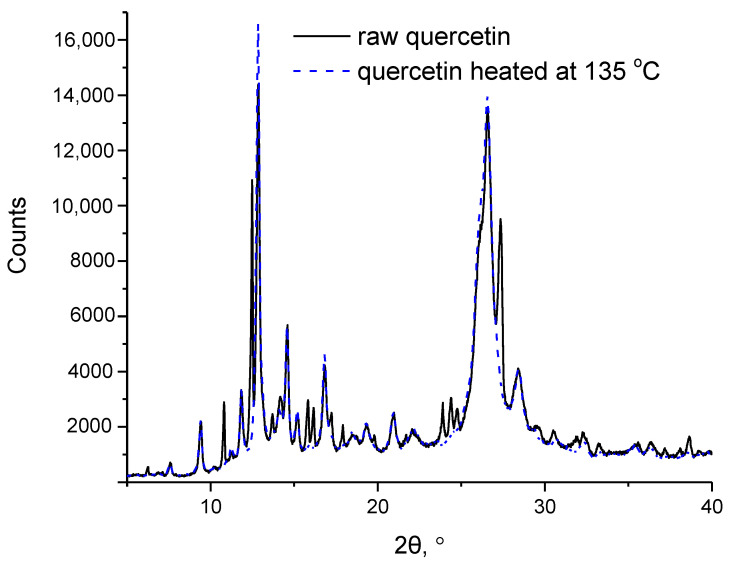
XRD patterns of raw quercetin and quercetin heated at 135 °C.

Quercetin prior to and after heating at 135 °C was also examined with XRD. The XRD patterns are presented in Figure 4. As can be seen in Figure 4, the overall pattern is similar for both samples, but some diffractions’ peaks have vanished, or their intensity has been strongly reduced after heating at 135 °C. This indicates that some distortion of the crystal lattice of quercetin has occurred. In combination with the alterations of the chemical structure revealed by FTIR, it is reasonable to conclude that the thermal effect of quercetin around 100 °C is a solid–solid thermochemical transition. A similar solid–solid thermochemical transition at around 90 °C has been very recently reported for gallic acid [15].

**Figure 5:**
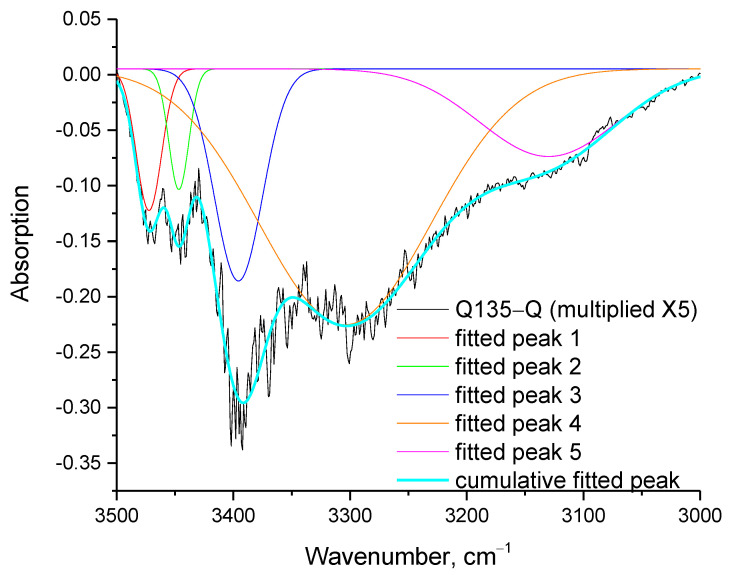
Fitting of the peak of the subtracted spectrum (Q135-Q) presented in Figure 3a in the range 3000–3500 cm^−1^ with 5 Gaussian peaks.

In order to further explore the phenomenon at 100 °C, a curve free fitting in the negative hydroxyl peak in the range of 3000–3500 cm^−1^ was performed with five Gaussian peaks, as shown in Figure 5. The wavenumbers and the percentage areas of each one of the five fitted peaks are presented in Table 2.

As can be seen in Table 2, the vast majority of OH groups that are missing in the heated sample are the OH groups that are shifted to lower wavenumbers, e.g., the 61% of the OH contributing to the negative peak appear at 3304 cm^−1^. In order for a proper fitting to be achieved, the peak with the lowest wavenumber should be centered at a wavenumber higher than 3100 cm^−1^. This suggests that in this peak, though there is undoubtedly a contribution from the =C-H groups, there is also a countable contribution from the highly shifted (bounded) OH groups. The high contribution of shifted OH groups can be explained as follows. The hydrogen bonding has a double effect on the thermochemical transition: (1) it constrains melting by keeping the molecules close to each other, due to specific strong intermolecular forces, and (2) it facilitates decomposition through the weakening of the chemical bonds.

Τhe equation for the vibrational wavenumber $\tilde{\nu }$ of the molecular oscillator is [30]:

$$\tilde{\nu }=\frac{1}{2\pi c}\sqrt{\frac{k}{\mu }}$$

where *c* is the speed of light, *k* is the force constant and *μ* is the reduced (effective) mass.

For the outliers of the OH region, that is 3550 cm^−1^ for the free phenolic and 3100 cm^−1^ for the highly shifted OH groups, the difference in the force constant of the free (*k_free_*) and of the shifted (*k_shifted_*) OH groups can be estimated from the above equation. The force constant is an established way to evaluate a chemical bond strength [31,32], and from the above calculation, it follows that within the structure of quercetin there are OH groups that may vary up to 24% in their bond strength. Of course, chemical bonds, besides molecular interactions such as hydrogen bonding, can weaken for other reasons [33], e.g., the presence of other chemical bonds and stereochemical factors. In any case, the results from the fitting (Table 2) confirm that the shifted hydroxyls, which are expected to be involved in the weak chemical bonds, have the highest contributions in the negative peaks of Figure 2 and Figure 5.

From the discussion in the Introduction section, it is clear that besides the qualitative confusion regarding the thermal transitions of quercetin, there is also a quantitative disagreement (the temperature values for the thermal transitions do not coincide in each study). Of course, the presence of impurities, as well as the heating rate used in the DSC measurement, can alter the measured temperature value. However, it is important to note that the quantitative disagreement regarding the exact temperature of the thermal transitions cannot be only attributed to such factors, since thermochemical transition most likely occurs in a rather broad temperature range. Thus, the quantitative disagreement for the temperatures of the thermal transitions can be considered to be of minor importance. In any case, in this study, a typical heating rate was used for the DSC measurement.

Besides the available literature data, e.g., for flavonoids quercetin dihydrate, rutin, silybin and similar molecules, e.g., gallic acid (see Introduction section), from the above discussion it can be suspected that other flavonoids and similar molecules with hydroxyl groups, e.g., tannins, are likely to exhibit similar peculiarities in their thermal behavior, that is solid–solid and solid–liquid thermochemical transitions. By the term “similar”, it is meant a molecule with a variety of chemical bonds and increased potential for hydrogen bonding. In other words, such high molecular weight compounds that are capable of simultaneously forming an increased number of hydrogen bonds, require increased thermal energy to break a vast amount of such strong specific intermolecular interactions in order to allow the softening and the subsequent melting. However, the absorption of such an increased amount of heat may also lead to a chemical bond rapture.

Finally, it is worth mentioning that the decomposition accompanied by some melting, dealt by Bawn kinetics, is actually a solid-liquid thermochemical transition with a high extent of decomposition, e.g., as it occurs for quercetin at 303 °C. As already mentioned, various substances exhibit qualitatively the same behavior, but with a low extent of decomposition. In some cases, this low extent of decomposition occurs at temperatures close to 100 °C and is not accompanied by softening. The low mass loss (low extent of decomposition) and the absence of softening typically leads to the conclusion that the detectable mass loss arises from water, or impurities, evaporation. However, this is not always the case, e.g., for gallic acid [15]. Besides these factors, the absence of mass loss after this first minor decomposition step for a broad temperature range (e.g., for silybin, no mass loss has been detected in the range 180–260 °C [14], or for quercetin as presented in the current study) also contributes to the confusion regarding the phase behavior of flavonoids and other pharmaceuticals. Regarding modelling and the difficulty in the prediction of the thermodynamic properties of such molecules, it can be concluded that besides the structure and intermolecular interactions, the strength of chemical bonds and their weakening due to various factors should be taken into account.

## 4. Conclusions

Quercetin does not exhibit any thermophysical transition such as a glass transition, nor neat melting. It exhibits two thermochemical transitions, one solid–solid transition at around 100 °C, and one solid–liquid transition that initiates at 150 °C and reaches its maximum at 303 °C. After 150 °C there is a continuous overlapping of absorbed heat and decomposition, leading to a structural relaxation. The low extent of decomposition during the thermochemical transition at around 100 °C is not enough to induce macroscopic fluidization, however the crystal lattice is distorted. Hydroxyl groups with shifted (lower) vibrational wavenumbers are involved in weaker chemical bonds and have the highest contribution to the minor decomposition at a temperature around 100 °C. In general, hydrogen bonding has a double effect on the thermochemical transition, i.e., it simultaneously prohibits melting and favors decomposition through the weakening of the chemical bonds. Similar behavior is expected by other flavonoids, and in general similar complex molecules with increased potential for hydrogen bonding, e.g., tannins with many (>2–3) hydroxyl groups. In such molecules, the heat required for melting (to loosen a vast amount of the hydrogen bonds) is comparable to the heat required for limited decomposition (to break some chemical bonds). The results of this study suggest that there is a potential connection among: (1) the variation in the reported experimental values of the thermodynamic properties of flavonoids, (2) the recognized difficulty in predicting the melting point and other similar thermodynamic properties in such molecules, and (3) the fact that Bawn kinetics, besides explosives, have been applied for pharmaceuticals. Such a connection should be the thermochemical transition, i.e., the structural relaxation occurring simultaneously with decomposition.

## Figures and Tables

**Table 1:** Heat measured by DSC, % mass loss from TGA and specific heat of fusion and thermochemical transition for the two endothermic peaks detected in the DSC curve of quercetin.

	Heat from DSC (mJ)	Mass Loss (%) from TGA	Specific Heat of Fusion	Specific Heat of Thermochemical Transition, J/g
Peak 1 (around 100 °C)	57.7	0.6–1.2	not applicable	1865–4110 *
Peak 2 (around 300 °C)	614.8	5.2	230–247 J/g 69–75 kJ/mol	4517

* This uncertainty arises from the uncertainty in the mass loss from TGA, but there are other more important sources of uncertainty. See text for details.

**Table 2:** Wavenumber and % area of the five fitted peaks presented in Figure 5.

Fitted Peak	Wavenumber, cm^−1^	% Area
1	3472	5
2	3447	3
3	3396	14
4	3304	61
5	3130	17

## Data Availability

Data are available upon request.

## Sample Availability

Samples of the compounds are available from the authors.
